# The placement of percutaneous retrograde acetabular posterior column screw based on imaging anatomical study of acetabular posterior column corridor

**DOI:** 10.1186/s13018-022-03347-3

**Published:** 2022-11-16

**Authors:** Kequan Yu, Runtao Zhou, Shichang Gao, Anlin Liang, Mingming Yang, Haitao Yang

**Affiliations:** 1 Orthopedic Laboratory and Department of Orthopedics, The First Affiliated Hospital of Chongqing Medical University, Chongqing, 400016 China; 2 Department of Radiology, The First Affiliated Hospital of Chongqing Medical University, Chongqing, 400016 China; 3 Department of Orthopedics, The Traditional Chinese Medicine Hospital of Yubei, Chongqing, 401120 China

**Keywords:** Acetabular posterior column, Retrograde screw, Imaging anatomical study

## Abstract

**Objective:**

To explore the entry point, orientation, and fixation range of retrograde acetabular posterior column screw.

**Method:**

The computed tomography data of 100 normal adult pelvises (50 males and 50 females, respectively) were collected and pelvis three-dimensional (3D) reconstruction was performed by using Mimics software and the 3D model was imported into Geomagic Studio software. The perspective of acetabular posterior column was carried out orienting from ischial tuberosity to iliac fossa in the Mimics software. Virtual screw was inserted perpendicular to the transverse section of acetabular posterior column corridor, and the maximum screw diameter, entry point, orientation, exit point were measured. The screw fixation range, the easy-to-penetrate sites, and intraoperative optimal fluoroscopic views were assessed.

**Results:**

The acetabular posterior column corridor showed a triangular-prism shape. The virtual screw entry point was located at the midline between the medial and lateral edges of the ischial tuberosity. The distance between the entry point and the distal ischial tuberosity was around 13 mm. The distances between the exit point and the true pelvis rim, and ipsilateral anterior sacroiliac joint line were (19.33 ± 2.60) mm and (23.65 ± 2.42) mm in males, respectively. As for females, those two data were (17.63 ± 2.00) mm and (24.94 ± 2.39) mm, respectively. The maximum diameters of screws were (17.21 ± 1.41) mm in males and (15.54 ± 1.51) mm in females. The angle between the retrograde posterior column screw and the sagittal plane was lateral inclination (10.52 ± 3.04)° in males, and that was lateral inclination (7.72 ± 2.99)° in females. Correspondingly, the angle between the screw and the coronal plane was anterior inclination (15.00 ± 4.92)° in males, and that was anterior inclination (12.94 ± 4.72)° in females. Retrograde acetabular posterior column screw through ischial tuberosity can fix the acetabular posterior column fractures which were not 4 cm above the femoral head center. The easy-to-penetrate sites were located at the transition between the posterior acetabular wall and the ischium, the middle of the acetabulum, and 1 cm below the greater sciatic notch, respectively. The iliac oblique 10°, iliac oblique 60°, and obturator oblique 60° views were the intraoperative optimal fluoroscopic views to assess whether the screw was safely inserted.

**Conclusion:**

Retrograde acetabular posterior column screw entry point is located at the midline between the medial and lateral edges of the ischial tuberosity, which is 1.3 cm far from the distal ischial tuberosity. The screw direction is about 10° lateral inclination and 15° anterior inclination, which can fix the acetabular posterior column fractures which were not 4 cm above the femoral head center.

## Background

Open reduction and internal fixation is the first choice for the treatment of displaced acetabular fractures; however, the operation is difficult and traumatic [1, 2]. Recently minimal invasive treatment of acetabular fractures has gained popularity, and percutaneous retrograde lag screw fixation has been shown to provide satisfactory results in noncomminuted, minimally displaced acetabular posterior column fractures, with the advantages of minimal surgical trauma, short operation time, little bleeding, and rigid fixation [3–5]. Because of the deep anatomical location of the acetabulum and its complex anatomical relationship with adjacent structures, improper operation during the surgery may penetrate the hip joint or injure the neurovascular bundles around posterior column, hence multiple C-arm imaging at different angles is required for safe intraosseous placement [6, 7].

Therefore, the present study using the Mimics software and Geomagic Studio software simulated the placement of percutaneous retrograde acetabular posterior column screw, and explored the morphological characteristics of acetabular posterior column corridor, the entry point, orientation, and easy-to-penetrate sites, which aimed to provide imaging anatomical evidence for retrograde lag screw in fixation of acetabular posterior column fractures.

## Materials and methods

### Data collection and semi-pelvic model building

The CT data of pelvis were collected from the department of radiology in the First Affiliated Hospital of Chongqing Medical University. From October 2017 to October 2018, 100 patients (50 women, 50 men) enrolled in the study, aged from 18 to 86 years old (mean age was 48.96 ± 18.48). Participators with tumor, fracture or infection were excluded. The data were reserved by digital imaging communication in medicine (DICOM) format. The DICOM format data were imported into Mimics 17.0 software (Materialise, Belgium) and 3D reconstruction of the pelvis was performed. The reconstructed 3D model of the pelvis was imported into the Geomagic Studio 2015 software (Geomagic, USA) in stereolithography (STL) format. The pelvis model was processed according to the method proposed by Feng et al. [8], and then imported into Mimics 17.0 software in STL format to match the original model.

### Parameters measurement of virtual screw

**Fig. 1:**
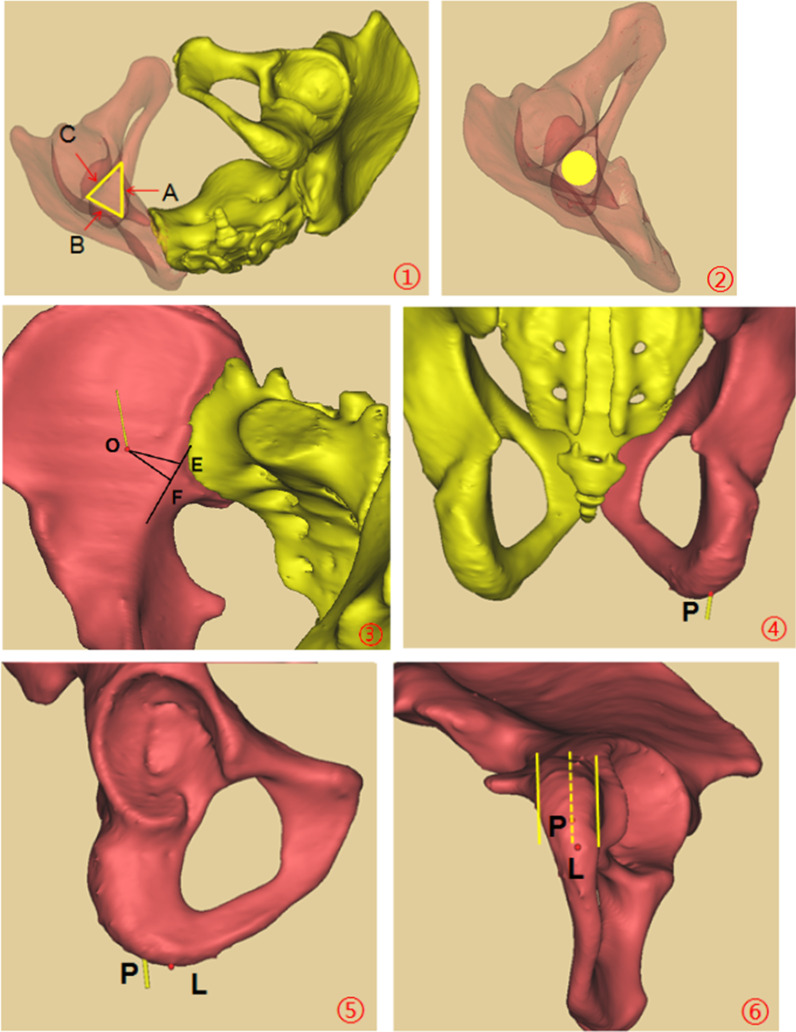
Parameters measurement of virtual screw. ① Axial perspective view of the right acetabular posterior column, the yellow triangle area is the safe zone for screw placement; A: Quadrilateral plate; B: Posterior acetabular wall; C: Acetabular dome. ② Axial perspective view of screw ③ Point O: Exit point; Line segment OF: Distance between exit point and the true pelvic rim; Line segment OE: Distance between exit point and ipsilateral anterior sacroiliac joint line. ④ Posterior view of the right hemipelvis; Point P: Entry point. ⑤ Lateral view of the right hemipelvis; Point P: Entry point; Point L: Most distal point of the ischial tuberosity. ⑥ Bottom view of the right hemipelvis; Point P: Entry point at the ischial tuberosity; The solid yellow lines: Medial and lateral edge of ischial tuberosity; The dotted yellow line: Central line of the ischial tuberosity

The perspective of acetabular posterior column was carried out orienting from ischial tuberosity to iliac fossa in the Mimics software. The acetabular posterior column corridor showed a triangular-prism shape, which was encircled by the posterior acetabular wall, the quadrilateral plate, and acetabular dome. Whereafter, the virtual screw was inserted perpendicular to the cross section of triangular prism. The maximum screw diameter was achieved by gradually increasing the screw diameter when the screw was tangent to the three sides of the cross section at the same time. We reduced the screw diameter to 0.5 mm, then the screw intersected the ischial tuberosity at Point P (entry point) and the medial cortex of the iliac fossa at Point O (exit point), respectively. The distance between Point P and O was the length of the acetabular posterior column corridor. The location relationship between Point P with the lateral and medial edge of ischial tuberosity was recorded. The distance between Point P and the distal ischial tuberosity was measured, which was recorded as PL. Passing Point O, the perpendicular line of true pelvic rim was drawn, and we obtained the intersection Point F. Passing Point F, the extension line of the true pelvic rim was drawn, and Point E was obtained which was the intersection of true pelvic rim extension line and the ipsilateral anterior sacroiliac joint line. The line segment OF was the distance between exit point and the true pelvic rim, and OE was the distance between exit point and ipsilateral anterior sacroiliac joint line (Fig. 1).

### Parameters measurement of acetabular posterior column corridor

**Fig. 2:**
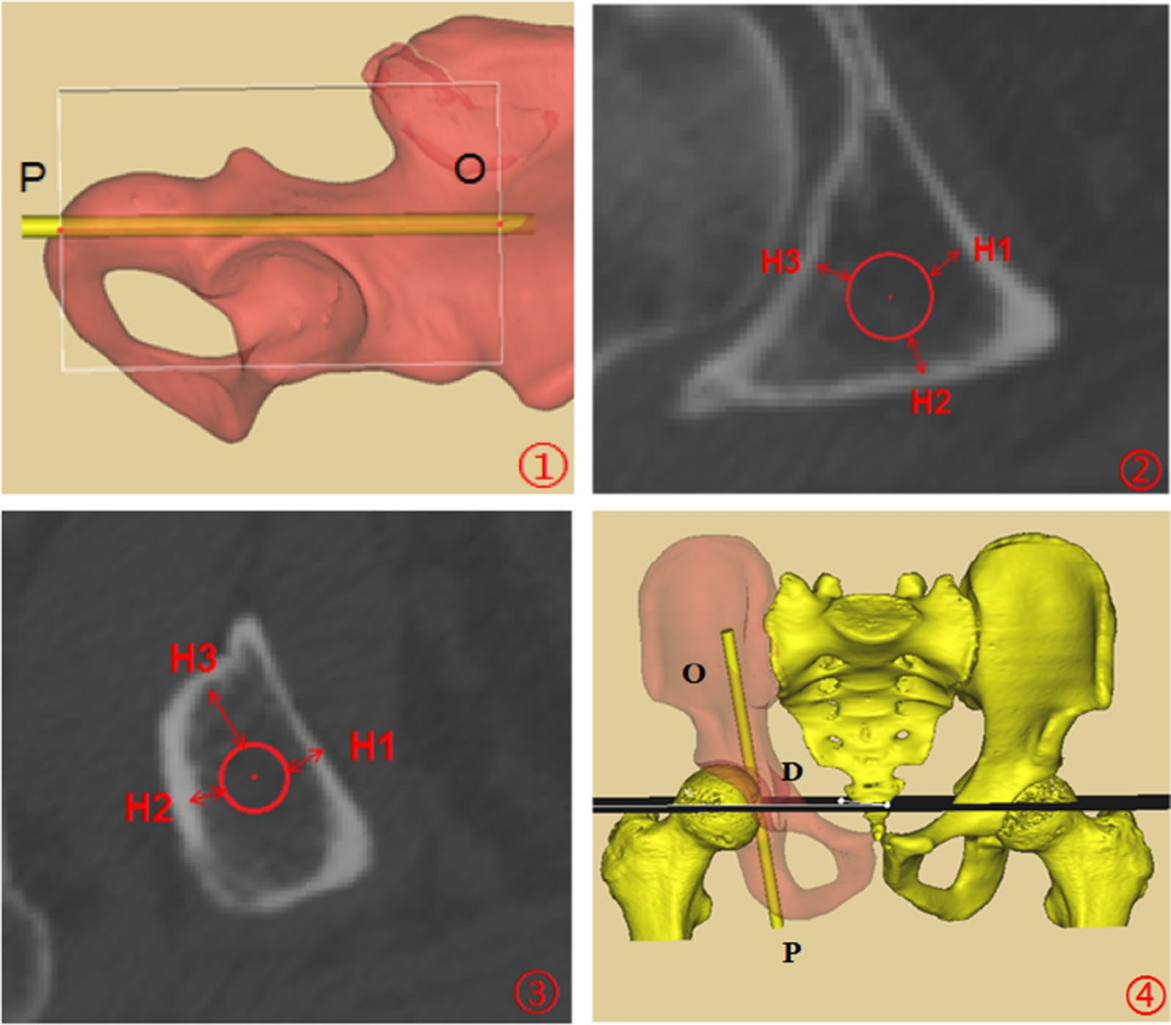
Parameters measurement of acetabular posterior column corridor. ① Re-cut range was from Point P to Point O; PO: Length of the acetabular posterior column corridor; ②, ③Different re-cut layers: H1: Distance between screw and quadrilateral plate; H2: Distance between screw and posterior acetabular wall; H3: Distance between screw and acetabular dome; ④Plane D: Horizontal plane of the femoral head center

**Fig. 3:**
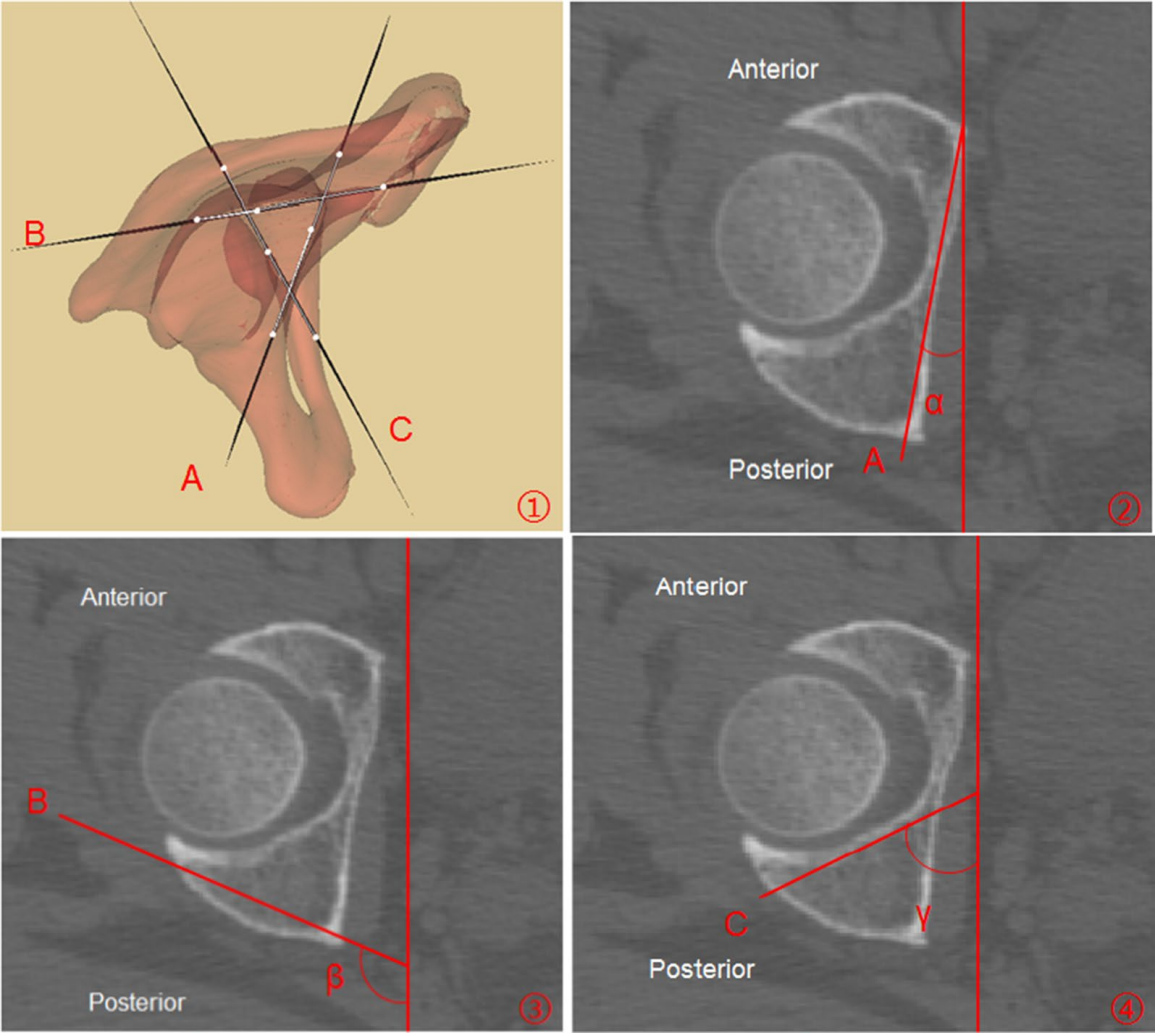
Measurement of angles between the sagittal plane and the three lateral faces of the triangular prism. ① Three lateral faces of the triangular prism: A: Quadrilateral plate; B: Posterior acetabular wall; C: Acetabular dome (According to the relevant anatomical measurements [3], the minimum cross section of the acetabular posterior column is mostly located at the level of the middle part of the acetabulum; therefore, at this level, the axial CT images were chosen to be measured.); ② A: Quadrilateral plate; α: Angle between quadrilateral plate and sagittal plane; ③ B: Posterior acetabular wall; β: Angle between posterior acetabular wall and sagittal plane; ④ C: Acetabular dome; γ: Angle between acetabulum and sagittal plane

1. Project function of the Mimics software was used to re-cut the acetabulum. The re-cut layers from Point P to Point O were perpendicular to the longitudinal axis of the screw with an interval of 5 mm.
2. The screw diameter was increased to 7.3 mm, then passing the border line of the screw in the sectional view, and the perpendicular lines of quadrilateral plate, posterior acetabular wall, and acetabular dome, respectively, were drawn. The distance between screw and quadrilateral plate, posterior acetabular wall, and acetabular dome were marked as H1, H2 and H3, respectively. We defined the high acetabular posterior column fracture as the fracture line was above the center of femoral head. Correspondingly, the fracture line of low acetabular posterior column fracture was below the center of femoral head. We measured the distance between the plane of femoral head center and 16 mm below the exit point, which was marked as DK, hence we could obtain the fixation range of screw (The screw was 16 mm threaded) (Fig. 2).
3. The angles between the Planes A, B, C and the sagittal plane on the pelvis in the standard anatomical position were measured and marked as *α*, *β*, and *γ*, respectively (Fig. 3).

### Measurement of virtual screw angle and safety range

**Fig. 4:**
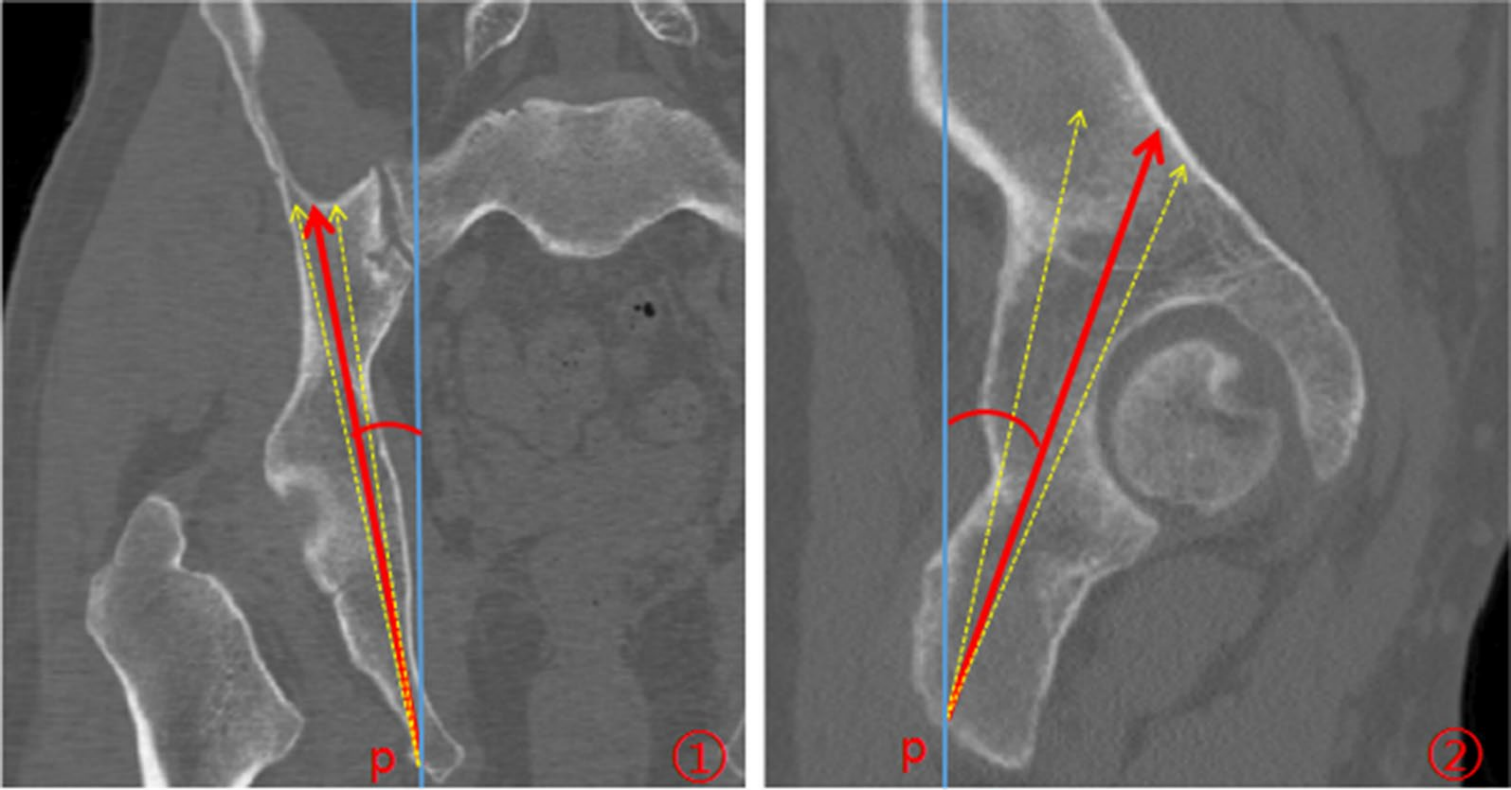
Measurement of screw angle. ① Point P: Entry point. The blue solid line represented the coronal plane; The red arrow represented the optimum orientation of the screw and the dotted yellow arrows on both sides represented the critical orientations. ② Point P: Entry point. The blue solid line represented the sagittal plane; The red arrow represented the optimum orientation of the screw and the dotted yellow arrows on both sides represented the critical orientations

We measured the angle between the virtual screw and the coronal and sagittal planes on the pelvis in the standard anatomical position and recorded it as the optimum screw angles. The Point P was fixed, and Point O was mobilizable, then screws (diameter = 7.3 mm) were just tangent to the cortex of the acetabular posterior column corridor, and the maximum screw angles with coronal and sagittal planes were obtained (Fig. 4.)

### Statistical analysis

All analyses were performed using SPSS software 19.0 (SPSS Inc., Chicago, Illinois). The differences of measurement data between male and female were compared with independent sample t test. The threshold for statistical significance was set at *p* value smaller than 0.05.

## Results

### Sex-specific differences in entry point location, length of corridor, and maximum screw diameter

The virtual screw entry point located at the midline is the ischial tuberosity. The distance between the entry point and the distal ischial tuberosity was (12.99 ± 1.99) mm in males. As for females, that was (13.26 ± 2.58) mm. According to statistical analysis, there was no significant difference between male and female. The distances between the exit point and the true pelvis rim, and ipsilateral anterior sacroiliac joint line were (19.33 ± 2.60) mm and (23.65 ± 2.42) mm in males, respectively. As for females, those were (17.63 ± 2.00) mm and (24.94 ± 2.39) mm, respectively. These differences had statistical significances between males and females. The corridor length and maximum screw diameter were significantly greater in males (141.59 ± 8.37 vs. 130.39 ± 6.30 mm, 17.21 ± 1.41 vs. 15.54 ± 1.51 mm, respectively) (Table 1).

**Table 1:** The comparison of diameter, entry point and exit point parameters of retrograde virtual acetabular posterior column screw between male and female (Mean ± SD, mm, *n* = 100)

	Diameter (mm)	PO (mm)	PL (mm)	OE (mm)	OF (mm)
Male	17.21 ± 1.41	141.59 ± 8.37	12.99 ± 1.99	23.65 ± 2.42	19.33 ± 2.60
Female	15.54 ± 1.51	130.39 ± 6.30	13.26 ± 2.58	24.94 ± 2.39	17.63 ± 2.00
*t*	5.700	7.532	− 0.571	− 2.680	3.675
*p*	< 0.001	< 0.001	0.570	0.009	< 0.001

### Features of acetabular posterior column corridor

**Fig. 5:**
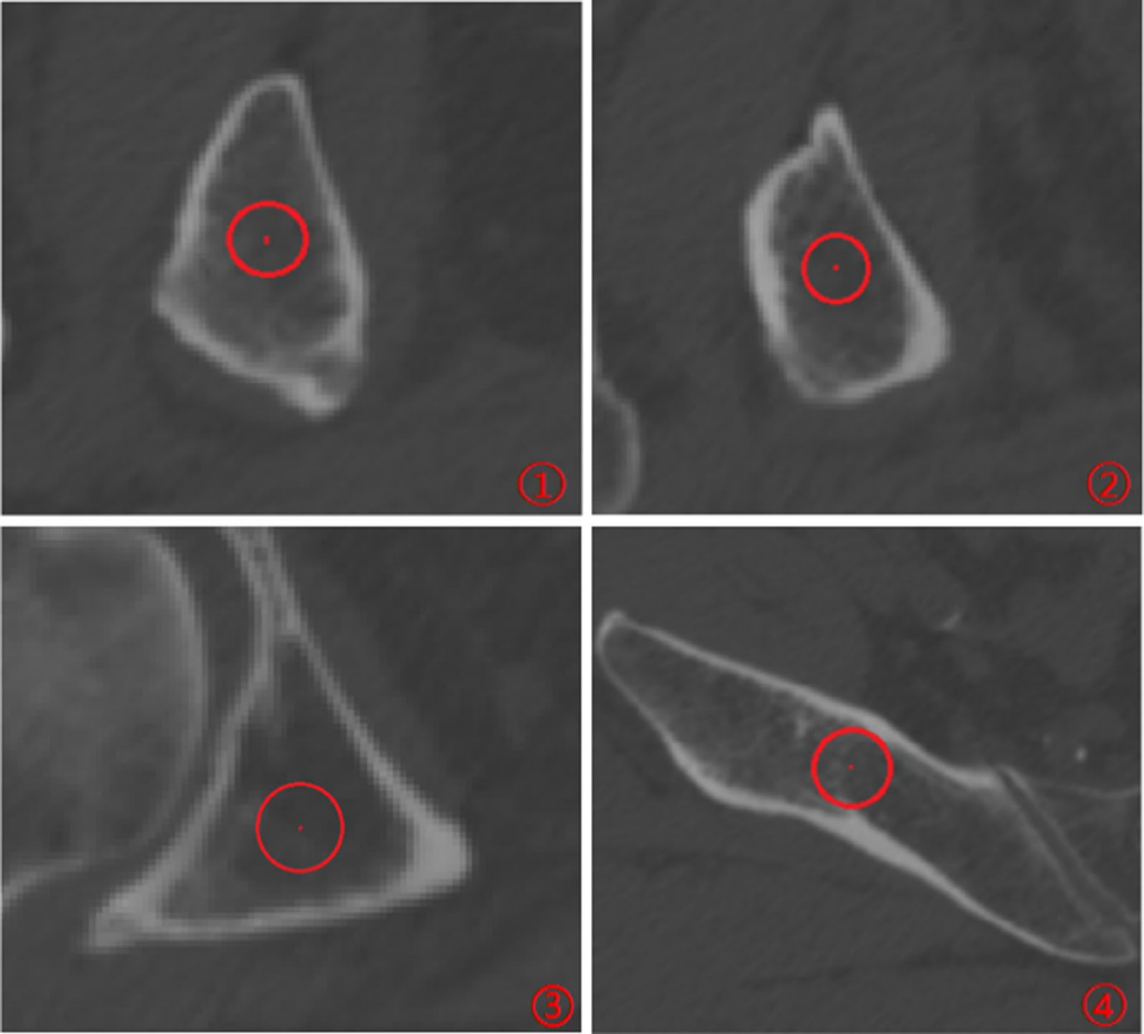
Morphological characteristics of different cross sections. ① Cross section of ischial tuberosity; ② Cross section between the transition of the ischial tuberosity and the inferior of acetabulum; ③ Cross section between the inferior of acetabulum and greater sciatic notch; ④ Cross section between greater sciatic notch and exit point

**Fig. 6:**
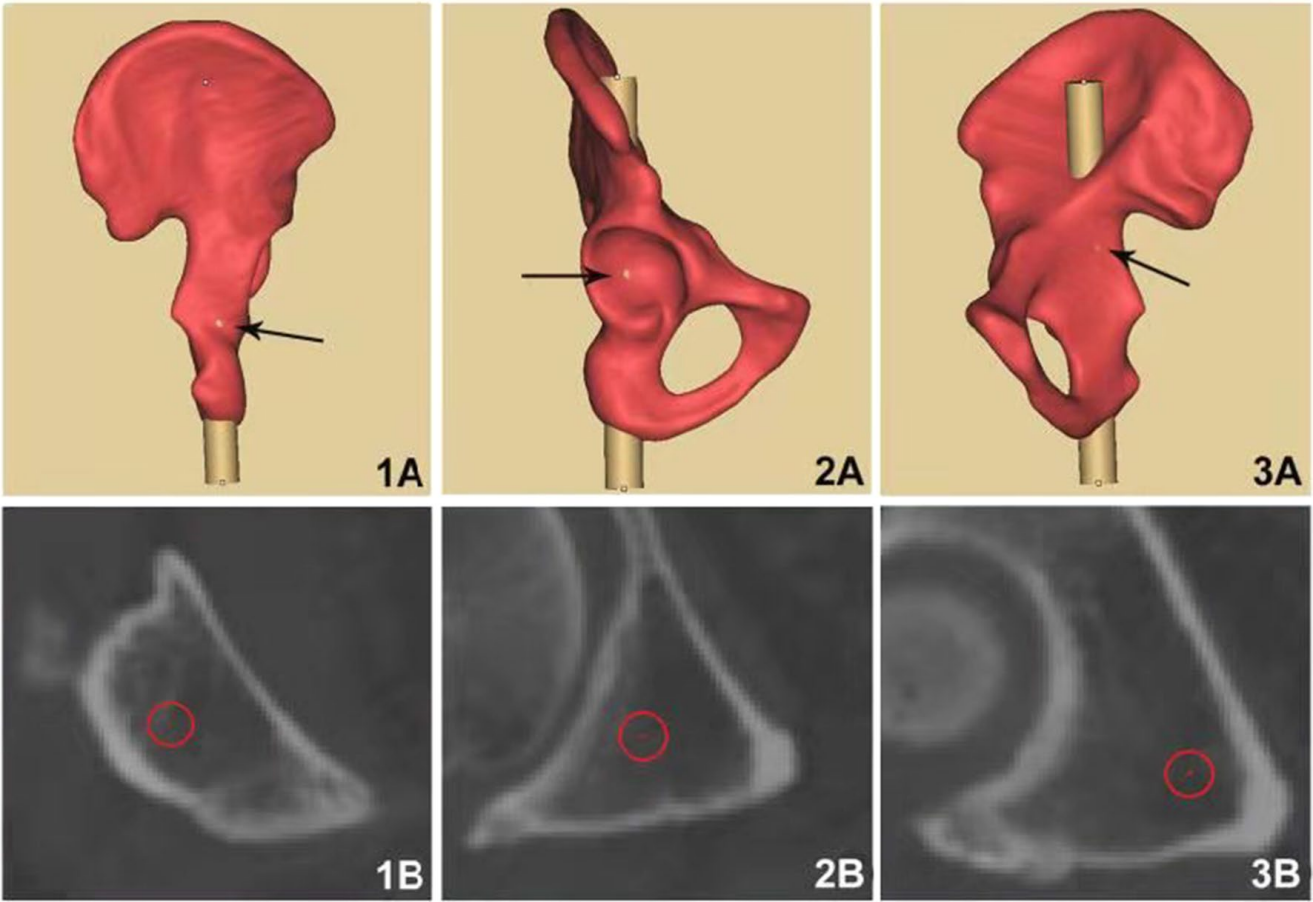
Cross sections of easy-to-penetrate sites. **1A** and **1B**: Posterior acetabular wall is easy-to-penetrate at 4th layer of cross section; **2A** and **2B**: Acetabulum is easy-to-penetrate at 7th layer in males or at 8th layer in females; **3A** and **3B**: Quadrilateral plate is easy-to-penetrate at 12th layer of cross section

Morphological characteristics of acetabular posterior column corridor were variable. There were 5–7 re-cut layers in the ischial tuberosity, and the shape of the cross section was approximate to triangle. From the transition of the ischial tuberosity to the inferior of acetabulum, there were just 2 layers. In this part, the cross section presented an oval shape. From the inferior of acetabulum to the greater sciatic notch, 10–12 layers were obtained, and the cross section shape converted to triangle again. There were 3–5 layers from the greater sciatic notch to exit point, and an approximate parallelogram shape of cross section was demonstrated (Fig. 5). The minimum distance between the screw and the posterior acetabular wall was (3.03 ± 0.90) mm in males and was (2.33 ± 1.03) mm in females, both of which were located at the 4th layer, approximately at the transition between the posterior acetabular wall and the ischium. The minimum distance between the screw and the acetabular dome was (2.63 ± 0.95) mm in males, which was at the 7th layer. As for females, that was (1.72 ± 0.87) mm, which was at the 8th layer. These two layers were around the middle of acetabulum. The minimum distance between the screw and the quadrilateral plate was (3.24 ± 0.94) mm in males and was (2.00 ± 0.76) mm in females, both of which were located on the 12th layer, approximately 1 cm below the greater sciatic notch (Fig. 6). According to statistical analysis, there were significant differences in the above data between male and female. At the same time, the retrograde screw through the ischial tuberosity could fix the low acetabular posterior column fractures and a part of high fractures. The fixation range of the high acetabular posterior column fracture, namely DK, was (45.92 ± 6.20) mm above the center of the femoral head in males and was (41.25 ± 4.20) mm in females, which had a significant difference between male and female (*t* = 4.406, *p* < 0.001). The *α*, *β*, and *γ* were, respectively (10.95 ± 2.29)°, (118.62 ± 4.43)°, and (63.62 ± 2.63)°in males, which were the angles between the three sides of the triangular-prism and the sagittal plane of pelvis. As for females, those were (11.73 ± 2.25)°, (119.39 ± 3.73)°, and (64.02 ± 2.69)°. The differences in these angles did not reach statistical significance between male and female. The above data are shown in Tables 2, 3, 4, 5.

**Table 2:** Comparison of the distance from screw to posterior acetabular wall between the male and female (Mean ± SD, mm, *n* = 100)

Layers	H2 (mm)		*t*	*p*	Layers	H2 (mm)		*t*	*p*
	Male	Female				Male	Female		
1	7.61 ± 2.21	5.11 ± 1.65	6.390	< 0.001	8	7.45 ± 1.75	5.35 ± 1.29	6.839	< 0.001
2	5.65 ± 2.37	4.84 ± 1.82	1.923	0.057	9	7.50 ± 1.74	5.78 ± 1.30	5.578	< 0.001
3	3.39 ± 1.54	3.94 ± 1.61	− 1.727	0.087	10	6.82 ± 1.70	5.55 ± 1.41	4.043	< 0.001
4	3.03 ± 0.90	2.33 ± 1.03	3.593	0.001	11	6.11 ± 1.53	4.98 ± 1.52	3.705	< 0.001
5	4.21 ± 0.88	2.43 ± 1.24	8.213	< 0.001	12	5.42 ± 1.48	4.07 ± 1.51	4.521	< 0.001
6	5.52 ± 1.12	3.30 ± 1.24	9.331	< 0.001	13	4.78 ± 1.40	3.42 ± 1.33	4.935	< 0.001
7	6.81 ± 1.38	4.49 ± 1.32	8.526	< 0.001

**Table 3:** Comparison of the distance from screw to acetabular dome between the male and female (Mean ± SD, mm, *n* = 100)

Layers	H3 (mm)		*t*	*p*	Layers	H3 (mm)		*t*	*p*
	Male	Female				Male	Female		
1	5.50 ± 1.81	4.41 ± 1.33	3.425	0.001	8	2.67 ± 0.90	1.72 ± 0.87	5.355	< 0.001
2	7.77 ± 3.87	4.88 ± 1.76	4.806	< 0.001	9	3.93 ± 0.96	2.53 ± 1.17	6.507	< 0.001
3	10.88 ± 1.75	6.73 ± 2.83	8.790	< 0.001	10	6.07 ± 1.14	4.13 ± 1.54	7.124	< 0.001
4	7.77 ± 1.45	7.77 ± 1.62	0.027	0.979	11	9.06 ± 1.26	7.19 ± 1.77	6.065	< 0.001
5	5.13 ± 1.19	5.40 ± 1.20	− 1.138	0.258	12	13.95 ± 1.41	12.26 ± 2.01	4.833	< 0.001
6	3.63 ± 0.97	3.32 ± 1.08	1.531	0.129	13	21.02 ± 1.18	19.46 ± 1.48	5.788	< 0.001
7	2.63 ± 0.95	2.18 ± 0.73	2.662	0.009

**Table 4:** Comparison of the distance from screw to quadrilateral plate between the male and female (Mean ± SD, mm, *n* = 100)

Layers	H1 (mm)		*t*	*p*	Layers	H1 (mm)		*t*	*p*
	Male	Female				Male	Female		
1	5.21 ± 0.90	3.20 ± 1.32	8.893	< 0.001	8	4.27 ± 0.98	3.69 ± 0.97	2.955	0.004
2	5.58 ± 0.83	4.00 ± 1.19	7.615	< 0.001	9	3.62 ± 0.96	3.08 ± 0.92	2.888	0.005
3	5.68 ± 0.89	4.40 ± 1.22	5.971	< 0.001	10	3.11 ± 0.99	2.67 ± 0.70	2.505	0.014
4	5.51 ± 0.89	4.70 ± 1.25	3.710	< 0.001	11	2.52 ± 0.93	2.23 ± 0.71	1.778	0.079
5	5.42 ± 0.93	4.84 ± 1.99	2.677	0.009	12	2.46 ± 0.87	2.00 ± 0.76	2.764	0.007
6	5.19 ± 0.95	4.63 ± 1.23	2.544	0.013	13	3.24 ± 0.94	2.44 ± 0.93	3.670	< 0.001
7	4.88 ± 1.05	4.29 ± 1.11	2.739	0.007

**Table 5:** Comparison of *α*, *β*, *γ* between male and female (Mean ± SD, *n* = 100)

	*α* (°)	*β* (°)	*γ* (°)
Male	10.95 ± 2.29	118.62 ± 4.43	63.62 ± 2.63
Female	11.73 ± 2.25	119.39 ± 3.73	64.02 ± 2.69
*t*	− 1.712	− 0.945	− 0.762
*p*	0.090	0.347	0.448

### Comparison of screw angle and safety range

We retrogradely inserted a 7.3 mm virtual screw through the ischial tuberosity. The angle between the retrograde posterior column screw and the sagittal plane was lateral inclination (10.52 ± 3.04)° in males, and (7.72 ± 2.99)° in females. Correspondingly, the angle between the screw and the coronal plane was anterior inclination (15.00 ± 4.92)° in males, and (12.94 ± 4.72)° in females. According to statistical analysis, there were significant differences between male and female. The combination of the two mean critical angles in the same plane presented the safety range of the screw. The safety range of lateral inclination was about 8°, and of anterior inclination was about 11° (Tables 6,7).

**Table 6:** Compare screw angles between male and female (Mean ± SD, *n* = 100)°

Lateral inclination angle (°)		Anterior inclination angle (°)
Male	10.52 ± 3.04	15.00 ± 4.92
Female	7.72 ± 2.99	12.94 ± 4.72
Mean	9.12 ± 3.31	13.97 ± 4.91
*t*	2.139	4.636
*p*	0.035	< 0.001

**Table 7:** Comparison of safety range between male and female (Mean ± SD, *n* = 100)

	Lateral inclination angle (°)			Anterior inclination angle (°)		
	Min	Max	Max–Min	Min	Max	Max–Min
Male	7.34 ± 3.33	15.94 ± 3.39	8.59 ± 0.23	9.85 ± 4.63	21.25 ± 5.49	11.40 ± 0.25
Female	4.86 ± 2.77	13.33 ± 3.04	8.47 ± 0.23	8.07 ± 5.03	19.02 ± 5.71	10.94 ± 0.31

## Discussion

### Anatomical characteristics of the acetabular posterior column corridor

**Fig. 7:**
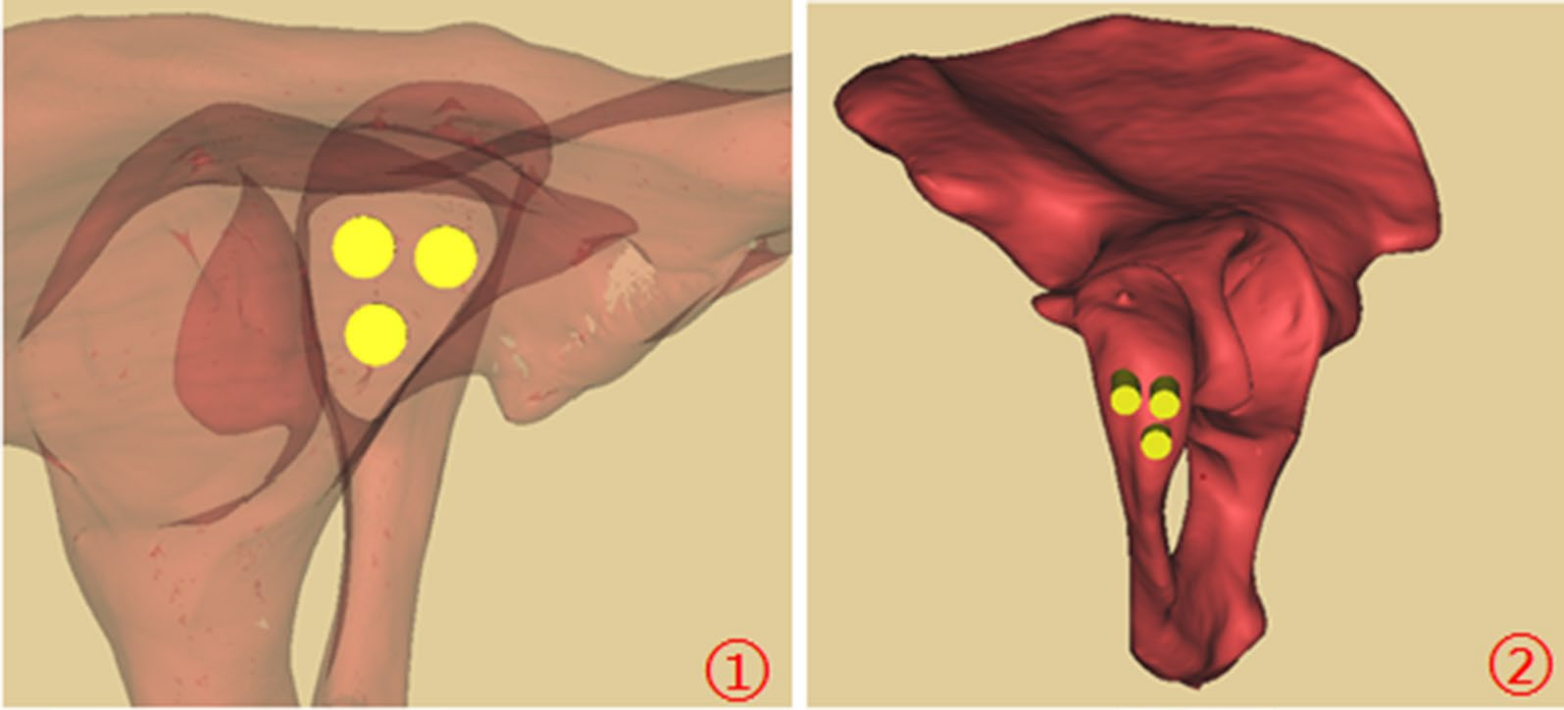
The acetabular posterior column is fixed by three lag screws

In our study, the length of acetabular posterior column corridor and the maximum screw diameter in males were greater than those in females, which might be caused by broader pelvis in males. The acetabular posterior column corridor demonstrated an irregular shape on the perspective, which had a triangular-prism shape from inferior of acetabulum to the greater sciatic notch. The diameters of acetabular posterior column corridor were 17.21 ± 1.41 mm and 15.54 ± 1.51 mm in males and females, respectively, which is sufficient to accommodate a virtual screw with a diameter of 7.3 mm. Furthermore, three 7.3 mm screws could be inserted in this corridor in males and three 6.5 mm screws were feasible for females in theory (Fig. 7), which suggested that there was enough space in the acetabular posterior column corridor to accommodate screws with larger diameters. Therefore, we recommended that a 7.3 mm screw can be used in both genders for the posterior column fixation.

What calls for special attention is that there were three sites with a high risk of penetrating the cortices, which were located at the transition between the posterior acetabular wall and the ischium, the middle of the acetabulum, and 1 cm below the greater sciatic notch, respectively. Correspondingly, when the screw penetrates the posterior acetabular wall at the transition between the posterior acetabular wall and the ischium, it is potential to damage the sciatic nerve; When the screw penetrates the cortex of middle acetabulum, the screw is easy to perforate the hip joint; Besides, there is a risk of damaging the superior gluteal nerve and vessels when screw penetrates the greater sciatic notch.

### Safe placement of acetabular posterior column screw

The placement of acetabular posterior column screw is greatly affected by the intraoperative body position. For the placement of antegrade posterior column lag screw, the ilioinguinal approach with patient supine is preferred. For the placement of retrograde posterior column screw through the ischial tuberosity, the screw can be inserted in prone or supine, even lateral position, with flexible hip and knee. The advantages of knee and hip joints flexion include straightforward access to the ischial tuberosity, relaxing sciatic nerve, and convenience of intraoperative fluoroscopy [5, 7, 9]. The virtual screw entry point located at the midline is the ischial tuberosity. The distance between the entry point and the distal ischial tuberosity was around 1.3 cm. The retrograde posterior column screw direction was lateral inclination (10.52 ± 3.04)° in males, and (7.72 ± 2.99)° in females. Correspondingly, the angle between the screw and the coronal plane was anterior inclination (15.00 ± 4.92)° in males, and (12.94 ± 4.72)° in females, which may be caused by the morphological differences of pelvis in genders. The pelvis is “funnel-shaped” in males, however that is “barrel-shaped” in females. At the same time, due to the individual difference of the screw insertion angle, an individual preoperative planning is recommended for each patient. Completing three-dimensional CT reconstruction of the pelvis, simulating the operation in Mimics system, and measuring the entry point and direction of screw will be helpful for the accurate individualized treatment.

**Fig. 8:**
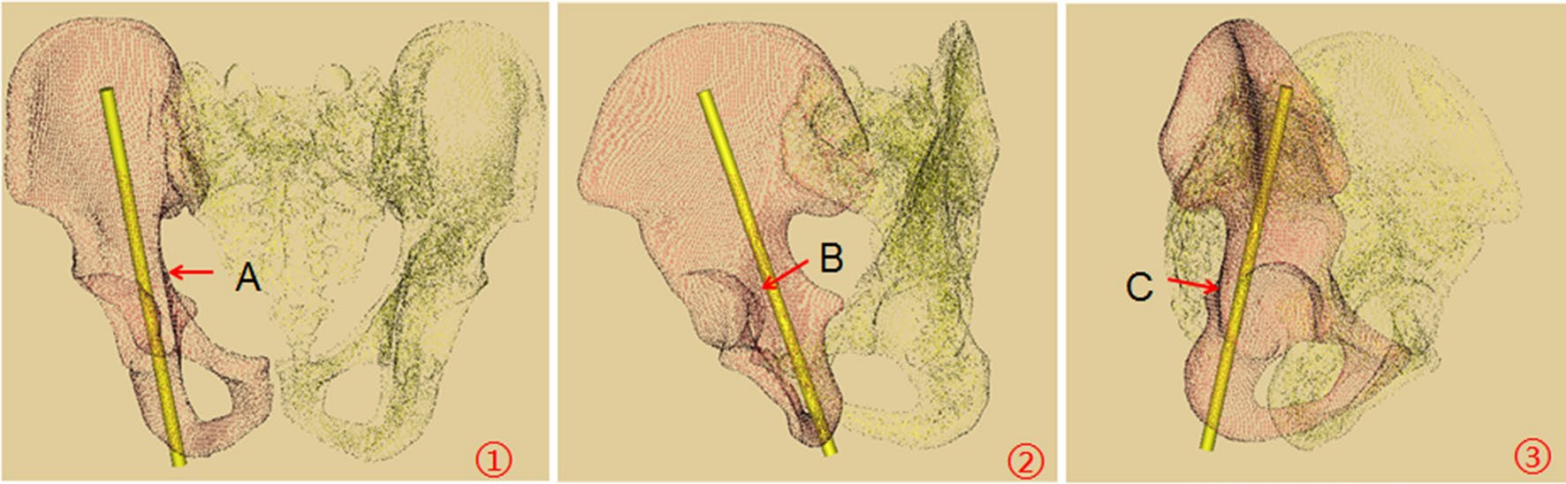
The optimal fluoroscopic views. ①The right iliac oblique 10° view; A: acetabular quadrilateral plate; ②The right iliac oblique 60°; B: acetabular dome; ③The right obturator oblique 60°; C: posterior acetabular wall

The pelvic anteroposterior view, iliac oblique and obturator oblique views are commonly used to verify whether the screw perforates the joint or cortex during the process of screw placement [7, 10, 11]. However, the acetabular posterior column structure is quite irregular, the above-mentioned fluoroscopic methods cannot fully reflect whether the screw penetrates the joint, the quadrilateral plate or the posterior acetabular wall. The posterior column is adjoining with sciatic nerve, superior gluteal nerve and vessels, and pelvic viscera; therefore a misdirected or misplaced screw during the surgery may cause serious complications [6, 12]. We found that the acetabular posterior column corridor is similar to the shape of a triangular prism, and the tangent positions of the three sides of the triangular prism can be used to assess whether the screw is safely inserted. When the ilium is obliquely positioned 10° for fluoroscopy, the quadrilateral plate can be roughly overlapped into a line. At this time, we can evaluate whether the screw penetrates the quadrilateral plate. In the same way, when the ilium is obliquely positioned 60°or the obturator foramen is obliquely positioned 60°, we can evaluate whether the screw penetrates the hip joint or posterior acetabular wall (Fig. 8). Wei Chen et al. [3] found that the posterior acetabular wall was abducted at 60° with the sagittal plane, and the quadrilateral plate was adducent at 9° with the sagittal plane. These results are similar to our study. Therefore, we can obtain the three tangent positions by moving the C arm according to the angles mentioned above, namely iliac oblique 10°, iliac oblique 60°, and obturator oblique 60° views, but further clinical verifications are needed. Nowadays, computer-assisted fluoroscopy-navigated percutaneous screw fixation technique has been advocated for its accuracy and versatility in acetabular posterior column fractures. However, this technique has often been frustrated in many hospitals primarily because of the high cost. In fact, the standard C-arm fluoroscopy is the most frequent image-guided technique in percutaneous screw fixation [13, 14].

### Selection of different acetabular posterior column screws

Acetabular posterior column screws were commonly used in clinics including long thread screws (32 mm threaded), partial thread screws (16 mm threaded), and full thread screws. When fixing acetabular posterior column fractures, the threaded head of the screw should completely traverse the fracture in order to achieve stable fixation; therefore the selection of screws may affect the fixation range. Biomechanical studies revealed that long threaded screws and fully threaded screws showed significantly higher stiffness and less failure compared to conventional partial threaded screws [15, 16]. In this study, we simulated the retrograde fixation of the acetabular posterior column with a virtual screw with a 16 mm thread, and we found that 16 mm threaded retrograde acetabular posterior column screws through ischial tuberosity can fix all the acetabular posterior column fractures which were not 4 cm above the femoral head center. Therefore, fully thread screws, long thread screws, or partial thread screws can be chosen to fix the low acetabular posterior column fractures, and all these kinds of screws can provide enough holding force. For high acetabular posterior column fractures, we can choose short-threaded screws in order to obtain a larger fixation range, but the risk of internal fixation failure caused by insufficient holding force should be taken into account. However, fully threaded screws can obtain the maximum fixation range and keep the stability of fixation. For fractures that are 4 cm above the femoral head center, antegrade screws are recommended for a reason that retrograde screws cannot provide enough holding force and stable fixation. In short, the fixation range of retrograde posterior column screws is affected by the type of screws, and the screw entry point and direction are quite vital for fracture fixation. Furthermore, preoperative Mimics virtual surgery and intraoperative computer navigation-assisted screw placement system can increase the safety and success rate of screw insertion, and reduce surgical risks and complications.

## Abbreviations

CT: Computed tomography
3D: Three-dimensional
DICOM: Digital imaging communication in medicine
STL: Stereolithography
